# Supplementary material for: Entwined African and Asian genetic roots of medieval peoples of the Swahili coast
Source: Nature. 2023 Mar 29;615(7954):866–73. doi: 10.1038/s41586-023-05754-w (PMC10060156; doi:10.1038/s41586-023-05754-w)
Supplement: Supplementary file 2 — Reporting Summary [file 41586_2023_5754_MOESM2_ESM.pdf]

nature portfolio

Corresponding author(s): Esther Brielle, Jeffrey Fleisher, Stephanie Wynne-Jones, Chapurukha Kusimba, David Reich

Last updated by author(s): Jan 11, 2023

Reporting Summary

Nature Portfolio wishes to improve the reproducibility of the work that we publish. This form provides structure for consistency and transparency in reporting. For further information on Nature Portfolio policies, see our Editorial Policies and the Editorial Policy Checklist.

Statistics

For all statistical analyses, confirm that the following items are present in the figure legend, table legend, main text, or Methods section.

n/a	Confirmed	
☐	☒	The exact sample size (*n*) for each experimental group/condition, given as a discrete number and unit of measurement
☐	☒	A statement on whether measurements were taken from distinct samples or whether the same sample was measured repeatedly
☐	☒	The statistical test(s) used AND whether they are one- or two-sided<br>*Only common tests should be described solely by name; describe more complex techniques in the Methods section.*
☐	☒	A description of all covariates tested
☐	☒	A description of any assumptions or corrections, such as tests of normality and adjustment for multiple comparisons
☐	☒	A full description of the statistical parameters including central tendency (e.g. means) or other basic estimates (e.g. regression coefficient) AND variation (e.g. standard deviation) or associated estimates of uncertainty (e.g. confidence intervals)
☐	☒	For null hypothesis testing, the test statistic (e.g. *F*, *t*, *r*) with confidence intervals, effect sizes, degrees of freedom and *P* value noted<br>*Give P values as exact values whenever suitable.*
☒	☐	For Bayesian analysis, information on the choice of priors and Markov chain Monte Carlo settings
☒	☐	For hierarchical and complex designs, identification of the appropriate level for tests and full reporting of outcomes
☒	☐	Estimates of effect sizes (e.g. Cohen's *d*, Pearson's *r*), indicating how they were calculated

Our web collection on statistics for biologists contains articles on many of the points above.

Software and code

Policy information about availability of computer code

Data collection	bwa-v0.6.1, OxCal version 4.4.2, contamMix version 1.0–12, IntCal20, SHCal20, Yfull version 8.09, HaploGrep2, https://github.com/DReichLab/ADNA-Tools and https://github.com/DReichLab/adna-workflow
Data analysis	ADMIXTOOLS version 7.0.2, Eigensoft version 7.2.0, ADMIXTURE version 1.23, DATES

For manuscripts utilizing custom algorithms or software that are central to the research but not yet described in published literature, software must be made available to editors and reviewers. We strongly encourage code deposition in a community repository (e.g. GitHub). See the Nature Portfolio guidelines for submitting code & software for further information.

Data

Policy information about availability of data

All manuscripts must include a data availability statement. This statement should provide the following information, where applicable:

- Accession codes, unique identifiers, or web links for publicly available datasets
- A description of any restrictions on data availability
- For clinical datasets or third party data, please ensure that the statement adheres to our policy

SNP array genotype data for 122 modern individuals newly reported in this study can be obtained from the Harvard Dataverse repository through the following link (https://doi.org/10.7910/DVN/NC28XW). BAM files of aligned reads can be obtained from the European Nucleotide Archive (accession no. PRJEB58698).

Human research participants

Policy information about studies involving human research participants and Sex and Gender in Research.

Reporting on sex and gender	The Madagascar samples were obtained from only male participants to allow joint analysis of nuclear genome variation and Y chromosome variation. The Emirati samples were obtained from any volunteers and gender or sex were not a factor in sample collection.
Population characteristics	For newly reported Madagascar genotype samples, individuals were chosen who would likely have a long-standing history in the region, namely older males with a maternal grandmother and a paternal grandfather also from the same area.<br><br>Emirati samples were collected from healthy adults as controls for a study on rare disease.
Recruitment	Individuals who participated in sample collection provided informed consent. and sampling strategies are as described above.
Ethics oversight	The Madagascar sampling was approved by Human Subjects' Ethics Committees of the Health Ministry of Madagascar and by French committees (Ministry of Research, National Commission for Data Protection and Liberties and Persons Protection Committee).<br><br>The Emirati sampling was approved by the Al Ain District Human Research Ethical Committee.

Note that full information on the approval of the study protocol must also be provided in the manuscript.

Field-specific reporting

Please select the one below that is the best fit for your research. If you are not sure, read the appropriate sections before making your selection.

☒ Life sciences ☐ Behavioural & social sciences ☐ Ecological, evolutionary & environmental sciences

For a reference copy of the document with all sections, see nature.com/documents/nr-reporting-summary-flat.pdf

Life sciences study design

All studies must disclose on these points even when the disclosure is negative.

Sample size	We did not predetermine sample size. Our analyses are based on the data we were able to successfully collect from ancient individuals, which is always difficult. Standard errors are reported to describe uncertainty ranges of our analyses that often reflects both sample size and data quality per sample.
Data exclusions	We exclude data with low coverage and data that comes from individuals that are first (or second) degree relatives of other individuals.
Replication	We observe similar ancestry patters among different ancient individuals from the same and different areas. We are also able to replicate sequence data from ancient individuals for which we have multiple libraries processed.
Randomization	We are unable to randomize historical events, and so we expect correlation between individuals. However, our formal modeling methods use a block jackknife to obtain a meaningful estimate of the uncertainty of our inference through analysis of uncorrelated segments of the genome each of whcih provides an independent view into past history.
Blinding	We contextualized our individuals within an archaeological framework in order to set up our analyses (such as setting up groups based on location and probable proxy source populations). Blinding was not applicable in this study since the archaeological context is important.

Reporting for specific materials, systems and methods

We require information from authors about some types of materials, experimental systems and methods used in many studies. Here, indicate whether each material, system or method listed is relevant to your study. If you are not sure if a list item applies to your research, read the appropriate section before selecting a response.

Materials & experimental systems

n/a	Involved in the study
☒	☐ Antibodies
☒	☐ Eukaryotic cell lines
☐	☒ Palaeontology and archaeology
☒	☐ Animals and other organisms
☒	☐ Clinical data
☒	☐ Dual use research of concern

Methods

n/a	Involved in the study
☒	☐ ChIP-seq
☒	☐ Flow cytometry
☒	☐ MRI-based neuroimaging

Palaeontology and Archaeology

Specimen provenance

Permits for collection and sequencing of each individual or group of individuals was obtained and described in the supplemental information as:

"Songo Mnara: Samples were selected from excavated skeletal remains at Songo Mnara. Samples from adult burials included 1-2 teeth for isotopic study as well as one metatarsal or metacarpal for aDNA analysis. Infants were not sampled during excavations. One infant mandible recovered during analysis of domestic faunal remains was also subjected to aDNA extraction. Teeth samples are stored at the University of Bristol. Bone samples are currently with the Reich laboratory at Harvard University; after this study is complete, they will be returned to Tanzania via the National Museums of Tanzania.
Kilwa and Lindi: For Kilwa and Lindi, samples were selected following protocols outlined by Prendergast and Sawchuk (2018) and Prendergast et al. (2019).

For Kilwa, three individuals were identified by E. Sawchuk among the commingled and largely unlabeled remains. Two samples were collected for one individual (KS.01.01, KS.01.02; the latter was not sampled and was returned intact), and one for each of the other individuals (KS.02.01, KS.03.01). Samples from Kilwa were exported and returned to the NMK by co-author C. Ogola in October 2017, and subsequently returned to the British Institute in East Africa (BIEA) where the rest of the remains are curated by co-author Dr. E. Ndiema in 2022. Arrangements have been made to repatriate all Kilwa human remains from the BIEA to the National Museum of Tanzania in Dar es Salaam.

For Lindi, two samples were chosen from the single individual present at this site, an adult male: the petrous portion of the left temporal bone (sample number LS1.02); and the right upper central incisor (LS1.01). Only the petrous was sampled, and the remainder of this petrous, and the complete unsampled incisor, were both returned to the NMT by M. Prendergast, along with a complete record of sampling activity and aDNA and C14 results, in May 2019. As described in a Memorandum of Agreement (MOA) between the Reich lab and the National Museum of Tanzania, the remaining powder, DNA extracts, and libraries remain under curation at the Reich Laboratory at Harvard University.

Mtwapa
Bone and tooth samples were taken over the course of the excavations at the site, which began under the direction of Kusimba in 1996. Most of the samples were collected by Monge and Williams during the 2010 season, however, when excavations focused on the cemetery located near the mosque. Tooth samples were collected for radiocarbon dating, stable isotope, and genetic analyses. Long bone fragments and rib samples were collected for stable isotope analyses. After each excavation season, the human remains were reburied, except for one individual remains are curated at the NMK. Any sample material remaining after analysis either has been or will be returned to Kusimba for curation at the NMK.

Manda
All samples were collected during a single excavation season in 2011-2012. Tooth samples were collected by co-authors J. Monge and S. Williams for radiocarbon dating, stable isotope, and genetic analyses. Long bone fragments and rib samples were collected for stable isotope analyses. The human remains were reburied the following year when osteological analyses were completed. Any sample material remaining after analysis either has been or will be returned to co-author C.M. Kusimba for return to the NMK.

Makwasinyi
Tooth fragment samples were collected by co-author C.M. Kusimba. The crania were left undisturbed in their respective localities. Any remaining sample material has been returned to co-author C.M. Kusimba for return to the NMK."

Specimen deposition

See above.

Dating methods

We provide new dates for several ancient individuals to understand the context of the admixture patters we detect and to help us build a chronology of these individuals. We obtained dating results from the Pennsylvania State University Radiocarbon laboratory or the Illinois State Geological Survey. Radiocarbon ages were calibrated "in OxCal version 4.4.2 (Bronk Ramsey (2009), using either the IntCal20 (Reimer et al 2020) or SHCal20 (Hogg et al. 2020) calibration latitude depending on whether the site was north (Kenya) or south (Tanzania) of the equator."

☒ Tick this box to confirm that the raw and calibrated dates are available in the paper or in Supplementary Information.

Ethics oversight

We obtained all permissions necessary for ancient DNA analysis from the respective countries from where the ancient individuals were buried or kept (see above), and we worked directly with communities that lived near the archaeological site and have traditions of inheritance from the ancient communities.

Note that full information on the approval of the study protocol must also be provided in the manuscript.
